# Chemical Composition and Source Apportionment of PM_2.5_ in Urban Areas of Xiangtan, Central South China

**DOI:** 10.3390/ijerph16040539

**Published:** 2019-02-13

**Authors:** Xiaoyao Ma, Zhenghui Xiao, Lizhi He, Zongbo Shi, Yunjiang Cao, Zhe Tian, Tuan Vu, Jisong Liu

**Affiliations:** 1 School of Resource, Environment and Safety Engineering, Hunan University of Science and Technology, Xiangtan 411201, China; maxiaoyao2002@163.com (X.M.); caoyj-xt@sohu.com (Y.C.); ljs930106@163.com (J.L.); 2 Atmospheric Environment Monitoring Station of Xiangtan, Xiangtan 411100, China; alphahe125@163.com; 3 School of Geography, Earth and Environmental Sciences, University of Birmingham, Birmingham B15 2TT, UK; z.shi@bham.ac.uk (Z.S.); tianzhe1129@gmail.com (Z.T.); v.vu@bham.ac.uk (T.V.); 4 Epsom Gateways, Atkins, Epsom KT18 5AL, UK

**Keywords:** PM_2.5_, chemical components, source apportionment, positive matrix factorization (PMF), Xiangtan City

## Abstract

Xiangtan, South China, is characterized by year-round high relative humidity and very low wind speeds. To assess levels of PM_2.5_, daily samples were collected from 2016 to 2017 at two urban sites. The mass concentrations of PM_2.5_ were in the range of 30–217 µg/m^3^, with the highest concentrations in winter and the lowest in spring. Major water-soluble ions (WSIIs) and total carbon (TC) accounted for 58–59% and 21–24% of the PM_2.5_ mass, respectively. Secondary inorganic ions (SO_4_^2−^, NO_3_^−^, and NH_4_^+^) dominated the WSIIs and accounted for 73% and 74% at the two sites. The concentrations of K, Fe, Al, Sb, Ca, Zn, Mg, Pb, Ba, As, and Mn in the PM_2.5_ at the two sites were higher than 40 ng/m^3^, and decreased in the order of winter > autumn > spring. Enrichment factor analysis indicates that Co, Cu, Zn, As, Se, Cd, Sb, Tl, and Pb mainly originates from anthropogenic sources. Source apportionment analysis showed that secondary inorganic aerosols, vehicle exhaust, coal combustion and secondary aerosols, fugitive dust, industrial emissions, steel industry are the major sources of PM_2.5_, contributing 25–27%, 21–22%, 19–21%, 16–18%, 6–9%, and 8–9% to PM_2.5_ mass.

## 1. Introduction

With rapid urbanization and industrialization in China, PM_2.5_ pollution has become one of the most important atmospheric related environmental issues. Extensive studies have shown that PM_2.5_ not only adversely affects air quality, visibility, and human health, but it also causes regional and global changes in climate [1,2,3]. Although the Chinese government issued the National Ambient Air Quality Standard of China (NAAQS—China) in 2012, more than 60% of megacities in the country do not yet meet the standard [1]. Water-soluble inorganic ions (WSIIs), especially secondary inorganic ions (SIAs: SO_4_^2−^, NO_3_^−^, and NH_4_^+^), were major chemical components of PM_2.5_ across China [1].

The Beijing—Tianjin—Hebei area (BTH), Yangtze River Delta (YRD), Pearl River Delta (PRD), and Sichuan Basin have the highest aerosol pollution levels in China, and many studies have been conducted in these regions to understand the general characteristics of the PM_2.5_ pollution and its chemical components, formation mechanism, and sources [4,5,6,7,8]. In addition, multiple studies have investigated the characteristics of atmospheric PM_2.5_ during episodes of fog and haze in several megacities in China [9,10,11].

**Figure 1:**
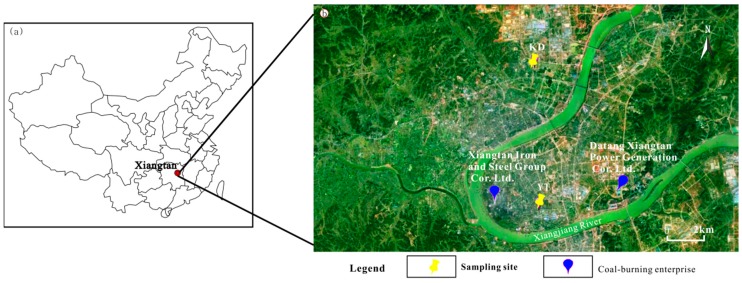
Location map of Xiangtan City (**a**) and satellite image showing the two sampling sites (**b**).

The city of Xiangtan, Hunan Province, is in the central southern region of China. The area is an intermountain basin within a subtropical climate zone and encircled by low and medium height hills with higher elevations to the north, west, and south (Figure 1). These features produce a year-round consistently high relative humidity and very low wind speeds.

Xiangtan plays an important role in the industrial base of Hunan Province and China as a whole, and although some enterprises have closed owing to industrial restructuring, several large enterprises (e.g., Xiangtan Iron and Steel Group Co. Ltd. and Datang Xiangtan Power Generation Co. Ltd.) remain in the urban area [12]. These source almost all of their energy needs from coal.

The chemical composition and source apportionment of PM_2.5_ aerosols under intermountain topographical and meteorological conditions should be of great interest to researchers; however, they have received little attention to date [12,13,14]. The results of the studies that have been conducted show that atmospheric PM_2.5_ pollution in Xiangtan City is relatively serious, especially in the winter [12,13]. Zhang et al. [13] showed that the volume of atmospheric particles and some heavy metals (e.g., Cd, Pb, and As) in winter exceed the national standard. Wang et al. [14] studied the regional distribution characteristics of polycyclic aromatic hydrocarbons (PAHs) in the PM_2.5_ to assess their risk to health. Tang et al. [12] studied the chemical compositions of the source apportionment of PM_2.5_ from Chang-Zhu-Tan city clusters (i.e., Changsha, Xiangtan, and Zhuzhou) and found that the mass concentrations of PM_2.5_ collected from September 2013 to August 2014 exhibited distinct regional differences (with the highest in Changsha and the lowest in Xiangtan) and seasonal variations (winter > autumn > spring > summer). However, according to the newest monitoring data (available from http://www.cnemc.cn), the daily mass concentrations are now higher in Xiangtan than in Changsha owing to the addition of PM_2.5_ pollution controls by local governments over the last five years. Based on this data, it is essential that the chemical composition and source apportionment of Xiangtan PM_2.5_ be studied further.

In this study, daily PM_2.5_ samples were collected simultaneously at two urban sites in the spring, autumn, and winter of 2016–2017, and various chemical components, including major water-soluble inorganic ions (WSIIs), carbonaceous species (i.e., OC (organic carbon) and EC (elemental carbon)), and metal elements were analyzed. The main objective was to characterize the seasonal and site differences of the PM_2.5_ chemical components to identify the major sources of PM_2.5_ particles and quantify their contributions.

## 2. Materials and Methods

### 2.1. Sampling Sites

The urban area of Xiangtan is divided by the Xiangjiang River, with the Yuetang and Yuhu Districts located to the east and west of the river, respectively (Figure 1). Daily PM_2.5_ samples were collected simultaneously at two urban sites, denoted YT and KD: the rooftop of the First Teaching Building on the South Campus of the Hunan Institute of Engineering in the Yuetang District (YT, 112°55′ E, 27°48′ N) and the rooftop of the Civil Engineering Building at the Hunan University of Science and Technology in the Yuhu District (KD, 112°55′ E, 27°54′ N). Samples were collected at sampling heights of approximately 18 and 20 m above the ground. The YT sampling site was chosen because of its close proximity to industrial, residential, and high-traffic areas of Xiangtan, including the locations of several important enterprises (e.g., Xiangtan Iron and Steel Group Co. Ltd.). In contrast, there is no significant industrial activity in the vicinity of the KD sampling site although nearby construction activities were ongoing.

### 2.2. Sample Collection

Daily (23 h) integrated PM_2.5_ samples were collected in three seasons: spring (28 April to 25 May 2016), autumn (12 September to 22 October 2016), and winter (2 December 2016 to 15 January 2017). Summer PM_2.5_ samples were not collected because of unusual and non-representative conditions. In order to be a ‘national civilized city’, in the summer of 2017 almost all of the buildings and roads in urban Xiangtan were renovated, and some large coal-burning enterprises had to reduce their emissions. At both sampling sites, PM_2.5_ samples were collected in parallel on quartz fiber filters (Whatman Inc., 90 mm, Piscataway, NJ, USA) to capture carbonaceous components and WSIIs, and polypropylene fiber filters (Whatman Inc., 90 mm) to capture mass and trace elements. PM_2.5_ sampling was conducted using two medium-volume samplers (TH-150C, Wuhan Tianhong Ltd., Wuhan, China) with a flow rate of 100 L/min.

Before sampling, the quartz filters were preheated to 550 °C for 4 h to remove any organic compounds. Before and after each sampling period, the bank and sample filters were equilibrated at a constant temperature (25 °C ± 1 °C) and relative humidity (40% ± 5%) for 48 h. During the sampling period, field blank filters were also collected by exposing the filters in the sampler without drawing air through them to account for any artefacts introduced during the sampling procedure. Once the collection period was complete, polypropylene fiber filters were immediately covered by tin paper and quartz fiber filters were stored in pre-baked aluminium foil and frozen at −18 °C until analyzed.

Meteorological parameters, including relative humidity (RH), wind speed (WS), temperature, and concentrations of SO_2_, NO_2_, CO, and O_3_, were measured hourly by co-located air quality monitoring stations operated by the Ministry of Environmental Protection in China.

### 2.3. Chemical Analysis

#### 2.3.1. Ions

Water-soluble ion concentrations were determined using an ion chromatography (IC) system (Dionex model ICS-3000, USA) as described by Xu et al. [15]. A quarter of each filter was cut into pieces and placed in 10 mL of ultrapure water (resistivity = 18.25 MΩ·cm) for 30 min to create an extraction solution, which was then filtered using a 0.22-μm pore syringe filter (Dionex Corp., Sunnyvale, CA, USA). The anion (F^−^, Cl^−^, SO_4_^2−^, and NO_3_^−^) concentrations were measured using an AS11-HC column (4 × 250 mm) with 30 mM KOH, while cation (NH_4_^+^, Na^+^, K^+^, Mg^2+^, and Ca^2+^) concentrations were determined using an Ion Pac CS12A column (4 × 250 mm) with 20 mM methane sulfonic acid as an eluent at a flow rate of 1.0 mL/min. Before conducting targeted sample analysis, a standard solution and blank test were performed; the correlation coefficient of the standard samples was more than 0.999. The detection limits were all lower than 0.03 mg/L. The recovery rates of the ions were in the range of 80–120%. All reported ion concentrations were corrected using field blanks.

#### 2.3.2. Carbon

The concentrations of organic carbon (OC) and elemental carbon (EC) on the quartz filters were measured using a Sunset Carbon Aerosol analyser (Sunset Laboratory Inc., Tigard, OR, USA) as described by Zhang et al. [16]. The organic carbon in the filter membranes was catalysed by manganese dioxide under the condition of no oxygen at 580 °C. Under the action of pyrolysis and fission, the carbonaceous combustion products were converted into carbon dioxide and then into an He/O_x_ mixture. Under aerobic conditions at 840 °C, methane gas was used as an internal standard during the entire measurement process to calibrate the FID response signal. A sucrose solution was used for external calibration to ensure sufficient measurement accuracy. All reported carbonaceous species concentrations were corrected using field blanks.

#### 2.3.3. Metals

The particles collected on the polypropylene fiber filters were analyzed for metal elements (i.e., Mg, Al, K, Ca, V, Cr, Mn, Fe, Co, Ni, Cu, Zn, As, Se, Mo, Cd, Sb, Ba, Tl, and Pb) via an inductively coupled plasma-mass spectrometer (ICP-MS; XSeries 2, Thermo Fisher, Waltham, MA, USA) as described by Liu et al. [17]. A quarter of the filters were digested in a high-pressure Teflon digestion vessel with a mixture of ultra-high purity acids (15 mL of HNO_3_ and 5 mL of HClO_4_) before being heated in a microwave system. The temperature of the microwave system was increased to 200 °C and maintained at that level for 30 min. Quality assurance and control were ensured through the analysis of certified reference material SRM 1649a (urban particulate matter); the standard reference material was pre-treated and analyzed using the same procedure. The resulting recoveries fell within ±10% of the certified values for most elements, except for Se, As, and Sb (±15%). All of the reported metal element concentrations were corrected using field blanks.

### 2.4. PMF Model

Positive matrix factorization (PMF), which was developed by Paatero and Tapper (1994) [18], is a receptor model that has been used to successfully identify the potential sources and source contributions without a priori knowledge of the profile of the local sources [5,12,18,19]. The PMF process for source apportionment of aerosol particles has been described in detail by many previous studies [18,19,20,21].

## 3. Results and Discussion

### 3.1. Mass Concentration of PM_2.5_

**Figure 2:**
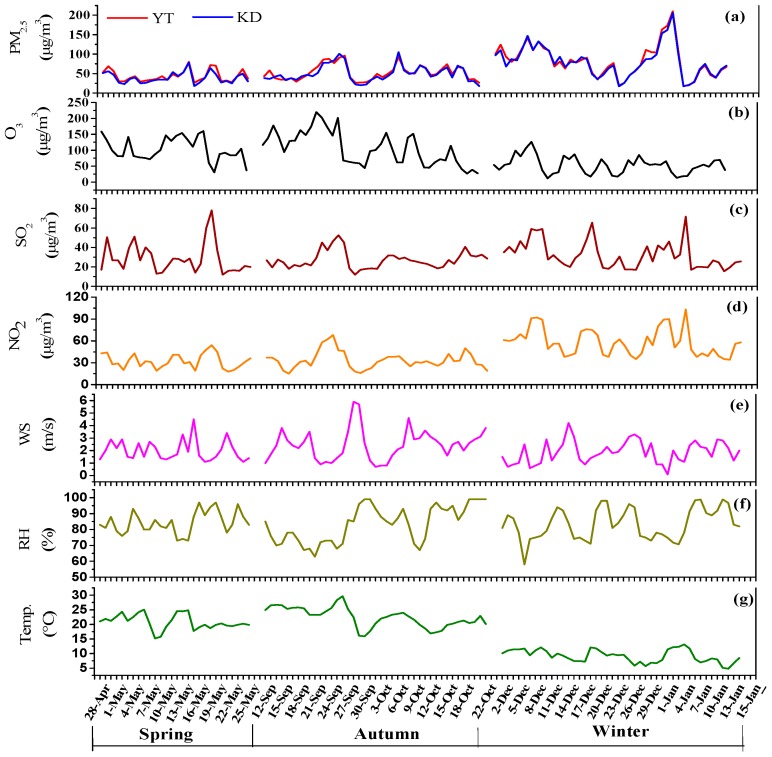
Concentrations of PM_2.5_, O_3_, SO_2_, and NO_2_, and meteorological data during the sampling periods: (**a**) PM_2.5_; (**b**) O_3_; (**c**) SO_2_; (**d**) NO_2_; (**e**) wind speed; (**f**) relative humidity; and (**g**) ambient temperature.

The concentrations of the PM_2.5_, O_3_, SO_2_, and NO_2_, and the meteorological data obtained during the sampling periods are shown in Figure 2 and Table 1. The mass concentrations of the PM_2.5_ during the sampling periods varied greatly within the range of 30–217 µg/m^3^. The seasonal variations in PM_2.5_ concentrations were significant and the mean concentrations were 93.4 ± 38.5, 82.2 ± 30.9, and 61.7 ± 18.5 µg/m^3^ at YT, and 95.6 ± 33.5, 68.2 ± 20.2, and 56.9 ± 19.3 µg/m^3^ at KD in winter, autumn, and spring, respectively. The spatial variations in the PM_2.5_ concentrations were not significant, which may indicate a similar regional pollution pattern for PM_2.5_ in Xiangtan. This suggests that a significant fraction of PM_2.5_ may consist of secondary particles [22,23]. In general, the concentrations of PM_2.5_ were lower than those of the Beijing—Tianjin—Hebei area (BTH) [6,10,24,25,26], Sichuan Basin [1,4], and Lanzhou [15], but were higher than Fuzhou [15] and Shenzhen [27].

As shown in Figure 2 and Table 1, the highest mass concentration of PM_2.5_ in winter was largely related to the combined effects of increased emissions (e.g., coal combustion for residential heating), confirmed by higher concentrations of SO_2_ and NO_2_ (Table 1), and unfavorable atmospheric diffusion conditions (i.e., low wind speeds and frequent temperature inversions). The low concentrations of PM_2.5_ in spring were mainly attributed to greater precipitation and more windy days, diluting and scavenging pollutants.

### 3.2. Chemical Compositions of PM_2.5_

#### 3.2.1. WSIIs

**Figure 3:**
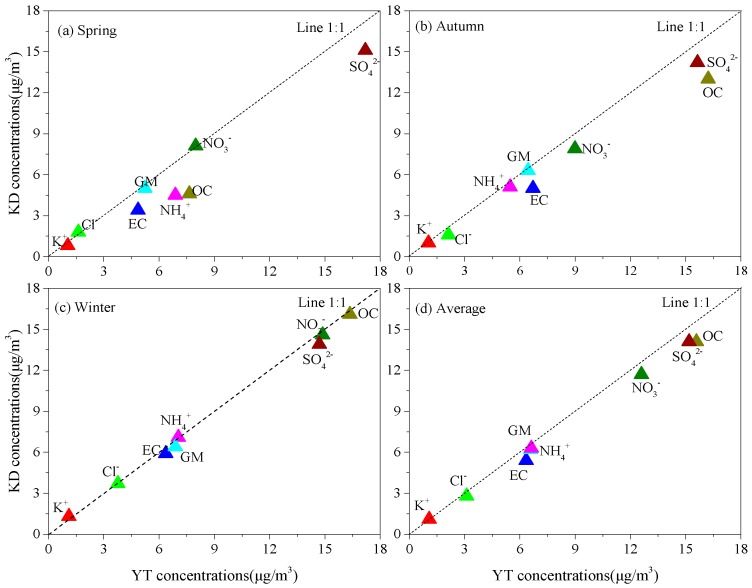
Seasonal mean concentrations of the major components of PM_2.5_ in (**a**) spring, (**b**) autumn, (**c**) winter, and (**d**) their average concentrations during the sampling periods at the YT and KD sites.

The mass concentrations of major WSIIs, their contribution to the PM_2.5_ concentration, and the sulphur (SOR) and nitrogen oxidation ratios (NOR) are shown in Table 2. The respective average concentrations of the total WSIIs were 44.6 ± 14.7 and 40.9 ± 12.6μg/m^3^ at YT and KD, accounting for 59.2% and 57.7% of the PM_2.5_ mass, respectively. The concentrations of the WSIIs were dominated by SO_4_^2−^, NO_3_^−^, and NH_4_^+^, followed by Cl^−^, with respective mean concentrations of 15.9 ± 4.7, 10.6 ± 6.1, 6.5 ± 2.7, 2.5 ± 1.0 μg/m^3^ at YT and 14.4 ± 4.3, 9.6 ± 5.0, 5.6 ± 2.7, 5.4 ± 1.5, 2.4 ± 1.0, and 1.2 ± 0.4 μg/m^3^ at KD. Secondary inorganic ions (SIAs: SO_4_^2−^, NO_3_^−^, and NH_4_^+^) accounted for 73.0% and 73.5% of the WSIIs at the two sites. From Table 2 and Figure 3, the mean concentrations of the total WSIIs exhibited distinctly seasonal variations and were highest in spring and lowest in winter. The concentrations of WSIIs were comparable with those from Chengdu and Chongqing [1,4], but higher than those in Lanzhou [25]. The concentrations of WSIIs and SIAs were lower than those in North China (e.g., Beijing—Tianjin—Hebei) and some cities (e.g., Nanjing and Heze) of East China, whereas the opposite was true for the corresponding percentage contribution to PM_2.5_ [10,19,20,26,28].

SOR and NOR can be used to evaluate the extent of the atmospheric conversion of SO_2_ to SO_4_^2−^ and NO_2_ to NO_3_^−^ as [24]

SOR = n(SO_4_^2−^) / [n(SO_4_^2−^) + n(SO_2_)], (1)

NOR = n(NO_3_^−^) / [n(NO_3_^−^) + n(NO_2_)]. (2)

As shown in Table 2, the SORs and NORs at the two sites during the sampling periods were all above 0.1, indicating that sulfate and nitrate were mainly produced by secondary transformation of SO_2_ and NO_2_ in the atmosphere [10,29]. Both the SOR and concentrations of particulate sulphate were highest in spring and lowest in winter at both sites, while the NOR and concentrations of particulate nitrates were highest in winter and lowest in spring.

**Figure 4:**
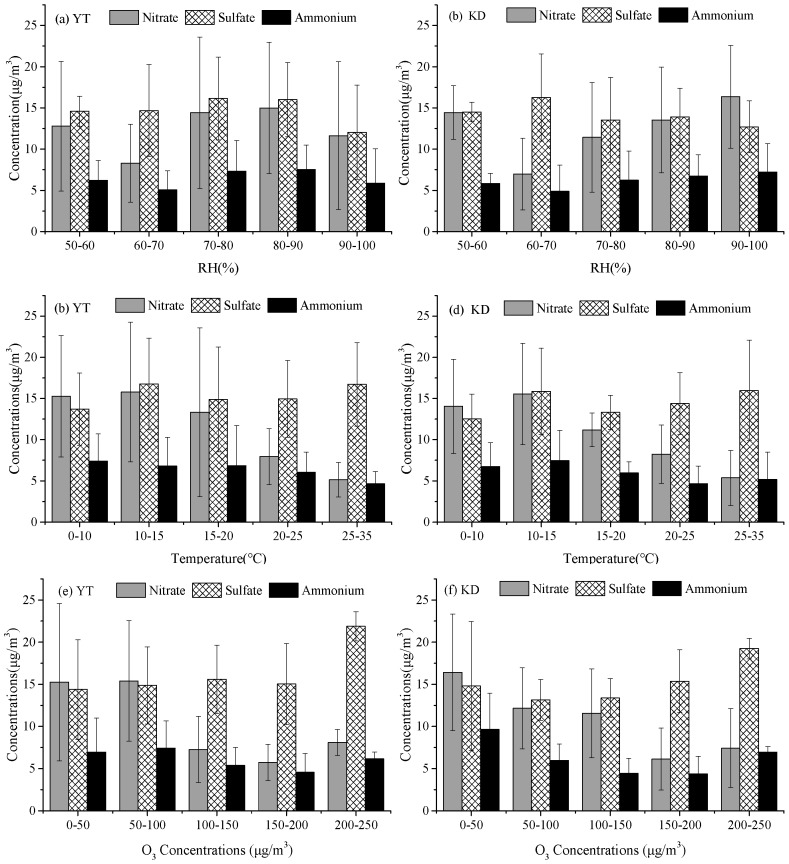
Comparisons of ions concentration in PM_2.5_ at the YT and KD sites for different ranges of meteorological factors: (**a**,**b**) relative humidity; (**c**,**d**) temperature; (**e**,**f**) O_3_ concentration.

Sulphate concentrations showed a poor correlation with relative humidity (Figure 4a,b), temperature (Figure 4c,d) and O_3_ concentrations (Figure 4e,f). The concentrations of nitrates are not correlated with relative humidity (Figure 4a,b) and O_3_ concentrations (Figure 4c,d) but decreases with increasing temperature. One of the possible reasons is that nitrate aerosol is less volatile at lower temperature [4,19]. The impact of meteorological conditions on sulfate and nitrate aerosol concentration is highly complex and the low time resolution of the samples (23 h average) makes it difficult to disentangle this effect.

It has been reported that the mass ratio of nitrate/sulphate can be used to evaluate the relative contributions of mobile and stationary sources in the atmosphere [15,30]. On average, the mass ratios of NO_3_^−^/SO_4_^2−^ at the two sites were less than one, especially in spring and autumn, which indicates that stationary sources make a greater contribution to aerosol pollution than vehicle exhaust [15,30]. It should be noted that the mass ratios of NO_3_^−^/SO_4_^2−^ at the two sites increased greatly from spring/autumn to winter (i.e., they were greater than one), which suggests that vehicle exhaust may have a greater contribution to PM_2.5_ in the winter. In addition, high atmospheric conversion of NO_2_ to NO_3_^−^ and a low atmospheric conversion of SO_2_ to SO_4_^2−^ may have contributed to the relatively high NO_3_^−^/SO_4_^2−^ values in the winter.

#### 3.2.2. Carbonaceous Species

The average mass concentrations of carbonaceous species at the two sites during the sampling periods were 13.4 ± 6.1 and 6.0 ± 1.8 μg/m^3^ at YT and 11.2 ± 5.2 and 4.8 ± 1.6 μg/m^3^ at KD (Table 3). The average total carbon (EC + OC) at YT accounted for 24.3% of the PM_2.5_ mass concentration during the sampling period, which was slightly higher than that at KD (21.1%). In addition, the OC concentration at the two sites during the sampling period exhibited strong seasonal variation, being highest in winter and lowest in spring. The concentrations of EC were higher in autumn and winter and lower in spring. Compared with OC, EC concentration exhibited less seasonal variability at the two sites, indicating a fairly uniform local source (e.g., primary particles from incomplete fossil fuel combustion). The OC and EC concentrations at Xiangtan were lower than those in Beijing—Tianjin—Hebei [7,24,26,28], Nanjing, and Heze [19,20], but higher than those in Chengdu and Chongqing [4].

Previous studies have shown that the OC/EC ratio is a potential indicator of the relative contributions of primary (POC) and secondary organic aerosols (SOC) [19,31]. Secondary organic carbon (SOC) is estimated as

(3) $$\mathrm{SOC}={\left(\mathrm{OC}\right)}_{\mathrm{tot}}-\mathrm{EC}\times {\left(\frac{\mathrm{OC}}{\mathrm{EC}}\right)}_{\mathrm{prim}}$$

where SOC (μg/m^3^) is the concentration of SOC, OC_tot_ is the total OC, and OC (μg/m^3^) and EC (μg/m^3^) are the concentrations of OC and EC. As reported by Wang et al. [4], (OC/EC)_min_ was simplified as (OC/EC)_prim_ to estimate SOC in this study. The estimated SOC was only an approximation; uncertainties mainly arise from the influence of biomass burning [4]. The results of this calculation are shown in Table 3. The OC/EC ratios in autumn and winter were all above 2.0. The seasonal patterns of SOC were observed to be similar to those of the PM_2.5_ and decreased in the order of winter > autumn > spring. The higher concentration of SOC in the winter is likely to be related to low temperature, favouring the condensation of semi-volatile organic aerosols [4,32].

#### 3.2.3. Metals

**Figure 5:**
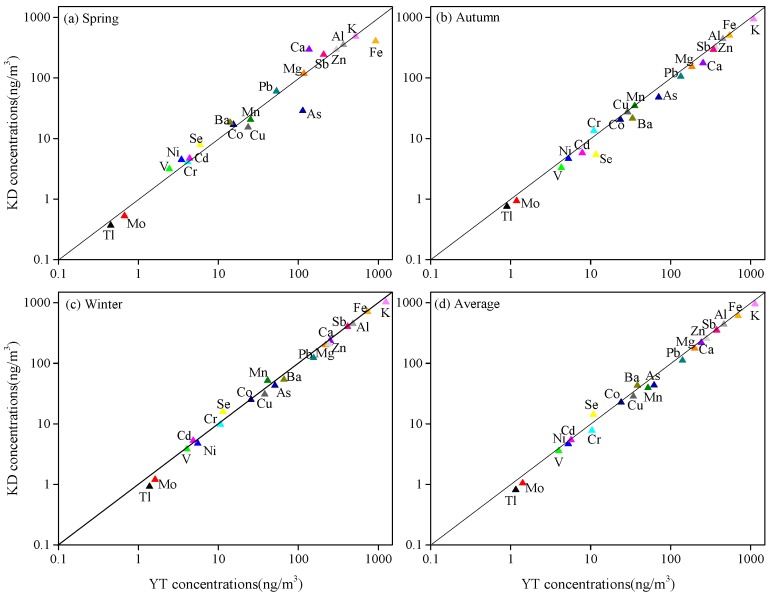
Seasonal mean concentrations of metal elements in spring (**a**), autumn (**b**), winter (**c**), and their average concentrations during the sampling periods (**d**) at the YT and KD sites.

The concentrations of metal elements in PM_2.5_ at YT and KD over the course of the sampling period are shown in Table 4 and Figure 5. The concentrations of K, Fe, Al, Sb, Ca, Zn, Mg, Pb, Ba, As, and Mn at both sites were higher than 40 ng/m^3^. The average concentrations of almost all detected metal elements at YT were higher than those at KD where the Xiangtan Iron and Steel Group Co. Ltd. is located (Figure 1); the exception was for Ca, Mn, and Se. The seasonal patterns of the detected metal elements (except Ca, Cr, Fe, Zn, As, and Cd) were also similar to that of the PM_2.5_ and decreased in the order of winter > autumn > spring. The seasonal patterns of Zn at the two sites decreased in the order of autumn> spring > winter; for Cd, the order was autumn > winter > spring. There were no uniform seasonal patterns for Ca, Cr, Fe, or As at either site.

**Figure 6:**
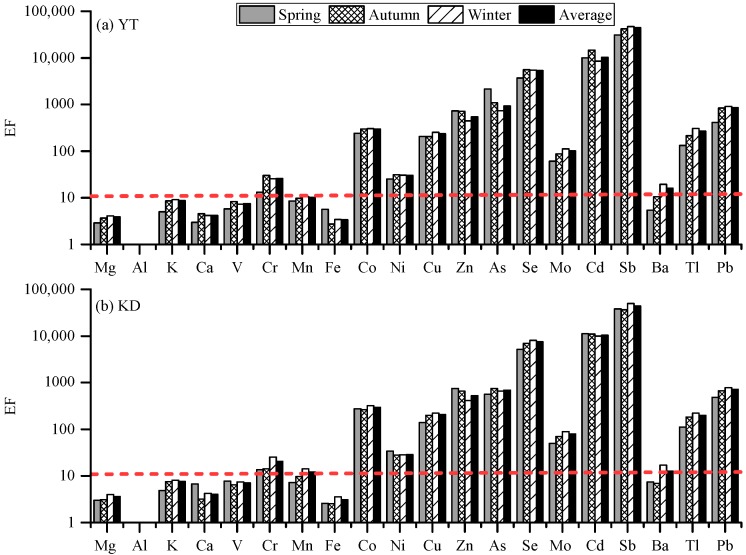
Enrichment factors (EF) values of the detected elements in the PM_2.5_ at the two sampling sites during the sampling periods ((**a**) YT, (**b**) KD).

To identify origins and evaluate the degree of anthropogenic influences, enrichment factors (EF) were calculated for the measured elements for each season. The calculation method is described in detail by Li et al. [19] and Liu et al. [20]. In this study, Al was used as the reference element [20]. The EF values of the detected elements in the PM_2.5_ at the two sampling sites during the sampling period are shown in Figure 6. During each season, the EF values for Mg, Al, K, Ca, V, Fe, and V at the two sites were all below 10, indicating that these metal elements may originate from crustal sources. The EF values for elements such as Cr, Ni, and Mo, were between 10 and 100, indicating a mixed (geological and anthropogenic) origin. In contrast, the EF values for Co, Cu, Zn, As, Se, Cd, Sb, Tl, and Pb at the two sites were all above 100, indicating an anthropogenic origin. For Mn and Ba, the EF values were higher than 10 in winter, suggesting a mixed origin. However, the EF values of Mn and Ba were less than 10 in the other seasons, indicative of crustal origin.

### 3.3. Source Apportionment Using PMF Models

**Figure 7:**
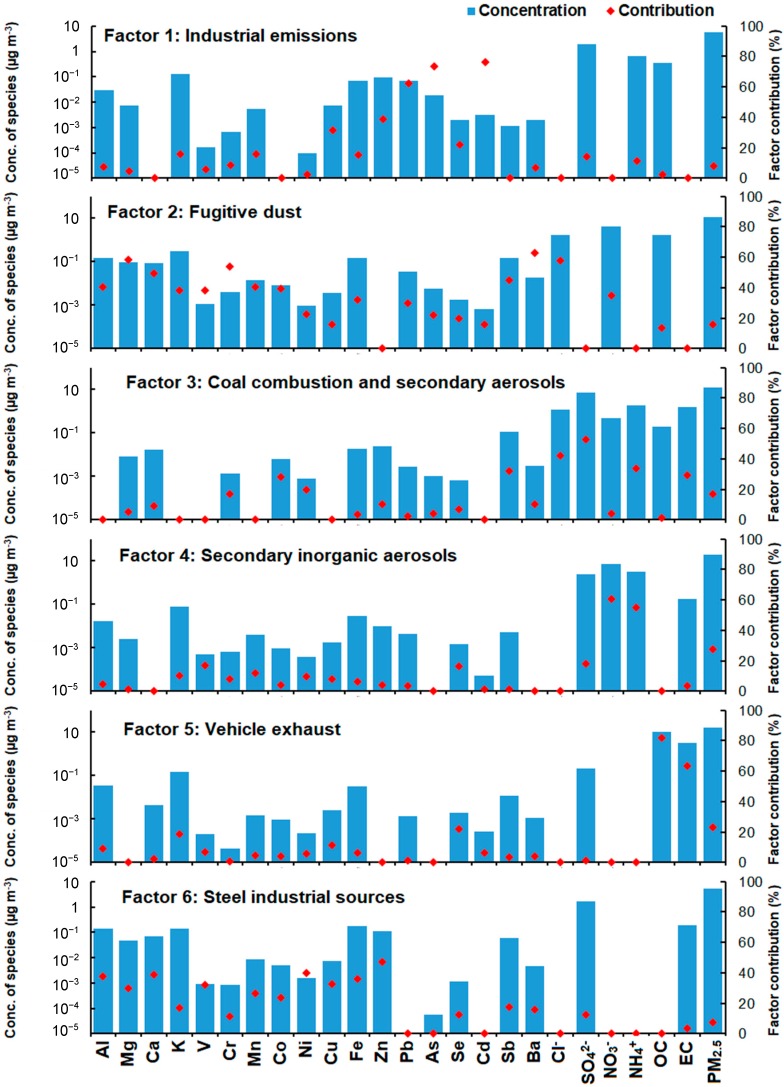
Factor profiles (bars and left y-axis) and percentage contributions (dots and right y-axis) of each chemical component resolved from the positive matrix factorization (PMF) model. Factor 1 to 6 represents industrial emissions, fugitive dust, coal combustion and secondary aerosols, secondary inorganic aerosols, vehicle exhaust, and steel industry.

In this study, 24 chemical components were used as inputs to the PMF model, including Al, Mg, Ca, K, V, Cr, Mn, Co, Ni, Cu, Fe, Zn, Pb, Cd, Sb, Ba, As, Se, Cl^−^, SO_4_^2−^, NO_3_^−^, NH_4_^+^, OC, and EC. In total, 20 runs were performed for each factor and the lowest value of Qrobust was 14,284.1 with a Qrobust/Qtrue ratio of more than 0.90. Six appropriate source factors were identified at both sites, representing industrial emissions, fugitive dust, coal combustion, secondary inorganic aerosol, vehicle exhaust, and steel industry. The factor profiles are shown in Figure 7.

**Figure 8:**
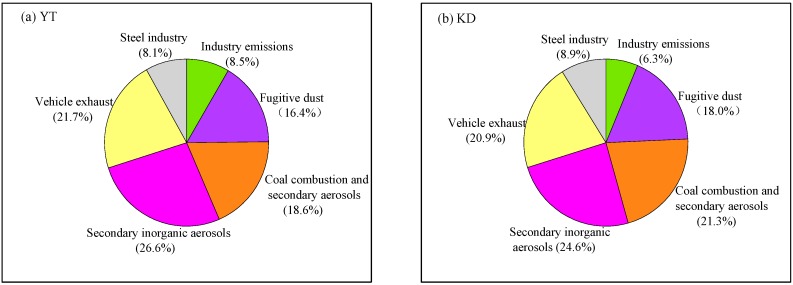
Contributions of different sources (factors) to PM_2.5_ mass at (**a**) YT and (**b**) KD.

Factor 1 in Figure 7 was associated with industrial emissions sources. The factor profile is characterized by a high load of Pb, Zn, Fe, and Cu, which are tracer elements of metal manufacturing plants and storage industrial emissions (e.g., Xiangtan Iron and Steel Group Co., Ltd.) [19,33,34]. In addition, other chemical components, such as Mn, Se, and Cd, also had high loadings for this source. An array of tracer species (Cr, Co, Cd, Zn, As, Fe, Cu, and Mn) have been used in India to identify specific industrial emissions [35,36]. Pb and Zn are major elements emitted from nonferrous metal smelting processes and from waste incinerators [37]. This factor contributed 8.5% and 6.3% to the PM_2.5_ mass at YT and KD sites (Figure 8).

Factor 2 in Figure 7 has been identified as fugitive dust (e.g., re-suspended dust), which show elevated loadings of Al, Ca, Fe, and Mg. The presence of Al, Ca, Fe, and Mg in PM_2.5_ from fugitive dust has been documented by many studies [5,6,20,33]. The EF values of the Al, Ca, Fe, and Mg were all less than 10, as shown in Figure 6, which further indicates that they primarily originated from crustal sources. This factor contributed 16.4% and 18.0% to the PM_2.5_ mass at YT and KD sites. As expected, this source contributed more at the KD site owing to a large area of arable land (Figure 1) and construction activities within the vicinity of the site.

Factor 3 in Figure 7 is likely to be associated with coal combustion and secondary aerosol (mixed sources). The factor is characterized by high loadings of SO_4_^2−^, Cl^−^, Sb and EC, typical of coal combustion; the relatively high loadings of NH_4_^+^ also suggests a contribution from secondary aerosols [5,6,19,20,21,38,39]. Some of the NH_4_^+^ may come from the after-treatment equipment for removing acidic gases from coal combustion; although the relatively high contribution of to NH_4_^+^ the profile suggests a non-negligible contribution from secondary sources to this factor. NH_4_^+^ is formed from gaseous NH_3_, which is emitted mainly from the agricultural sector (most notably animal manure and fertilizer application) [19,40]. This factor contributed 18.6% and 21.3% to the PM_2.5_ mass at YT and KD sites (Figure 8). According to the Xiangtan Statistical Yearbook 2016, the total amount of coal consumed in Xiangtan was ~8129 million tons in 2016, which accounted for about 70% of the total energy consumption.

Factors 4 in Figure 7 were identified as secondary inorganic aerosols, which were characterized by high loads of NO_3_^−^, NH_4_^+^, and SO_4_^2^^−^. This factor contributed 26.6% and 24.6% to the PM_2.5_ mass at YT and KD sites (Figure 8). Previous studies have found that these inorganic ions are markers of secondary inorganic aerosols [5,6,19,20,21,31], and as discussed, are often formed by heterogeneous and homogeneous processes under favourable meteorological conditions [4,10,41,42,43,44]. NO_3_^−^ is mainly converted from ambient NO_x_ emitted by both vehicle exhausts and fossil fuel combustion, while the precursor of aerosol SO_4_^2−^ is SO_2_, which primarily originates from coal combustion [1,32]. Therefore, the actual contributions of coal combustion and vehicle exhaust to PM_2.5_ mass discussed above were likely underestimated.

Factor 5 in Figure 7 is likely to be from vehicle exhaust, which has high loadings of OC and EC. This factor contributed 21.7% and 20.9% to the PM_2.5_ mass at YT and KD sites (Figure 8). The presence of OC and EC in the PM_2.5_ from vehicle exhaust has been documented previously [6,20,21,37].

Factor 6 in Figure 7 were associated with steel industry sources, which were characterized by a high load of Fe. In addition, other chemical components, such as Zn, Cu, and Pb, also had high loadings for this source. The attribution of Fe, Zn, Pb, in PM_2.5_ to the iron and steel industry has been documented by many other studies [19,33,34]. This factor contributed 8.1% and 8.9% to the PM_2.5_ mass at YT and KD sites (Figure 8).

Overall, secondary inorganic aerosols (25.7%) was found to be the largest contributor to PM_2.5_ at Xiangtan city, followed by vehicle exhaust (21.3%), coal combustion and secondary aerosols (19.9%), fugitive dust (17.1%), steel industry (8.5%), and industrial emissions (7.5%).

Based on the results of source apportionment, we recommend that emissions due to vehicle exhaust and coal combustion should be the priority targets to reduce the PM_2.5_ pollution in Xiangtan. This will not only reduce the primary emissions but also the secondary aerosols formed from SO_2_ and NO_x_.

## 4. Conclusions

In this study, seasonal and spatial variations as well as the potential sources of PM_2.5_ collected in two urban areas of Xiangtan, central south China, were investigated. The mass concentrations of PM_2.5_ during the sampling periods were in the range of 30–217 µg/m^3^, being highest in winter and lowest in spring.

The mean concentrations of WSIIs were 44.6 ± 14.7 and 40.9 ± 12.6 µg/m^3^ at YT and KD sites, respectively, accounting for 59.2 ± 9.8% and 57.7 ± 10.4% of the PM_2.5_ mass, respectively. The WSIIs were dominated by secondary inorganic ions (i.e., SO_4_^2−^, NO_3_^−^, and NH_4_^+^), which accounted for 43.5 ± 8.3% and 42.6 ± 8.5% of the PM_2.5_ mass concentration at YT and KD, respectively. The highest concentrations of SO_4_^2−^ and SOR at the two sites occurred in the spring while the lowest were in winter. These findings differ from those for NO_3_^−^ and NOR. The average concentrations of total carbon (EC + OC) were 19.4 ± 7.8 and 16.0 ± 6.8 µg/m^3^ at YT and KD, accounting for 24.3% and 21.1% of the PM_2.5_ mass, respectively. The concentrations of K, Fe, Al, Sb, Ca, Zn, Mg, Pb, Ba, As, and Mn in PM_2.5_ at the two sites were relatively high (more than 40 ng/m^3^). EF values for Mg, Al, K, Ca, V, Fe, and V at the two sites were all below 10, which indicates that they may be primarily originated from crustal sources.

Six factors were identified by PMF at Xiangtan, representing secondary inorganic aerosols, vehicle exhaust, coal combustion and secondary aerosols, fugitive dust, industrial emissions, and steel industry. The first three sources are the dominant ones, contributing over 67% to PM_2.5_ mass. Thus, it is recommended that secondary inorganic aerosols, coal combustion, and vehicles are the primary targets in order to reduce PM_2.5_ pollution in Xiangtan.

## Figures and Tables

**Table 1:** Average concentrations of SO_2_, NO_2_, and O_3_ in the atmosphere and average temperature, relative humidity, and wind speed during the sampling periods.

Pollutant and Meteor. Parameters	YT				KD			
	Spring	Autumn	Winter	Average	Spring	Autumn	Winter	Average
O_3_	134.4 ± 10.6	129.4 ± 55.8	62.6 ± 30.4	108.8 ± 32.3	129.5 ± 32.5	126.0 ± 10.9	58.4 ± 24.8	104.6 ± 22.7
SO_2_	27.6 ± 13.5	31.0 ±10.6	33.3 ± 13.8	30.6 ± 12.6	26.1 ± 12.7	30.7 ± 14.5	33.6 ± 12.2	30.1 ± 13.1
NO_2_	37.6 ± 7.1	41.1 ± 13.4	52.4 ± 19.9	43.7 ± 13.5	36.5 ± 12.5	39.7 ± 12.9	59.2 ± 18.1	45.1 ± 15.2
T (°C)	20.8 ± 1.1	22.1 ± 6.0	9.5 ± 3.7	17.4 ± 3.6	20.6 ± 1.2	22.1 ± 6.6	9.1 ± 2.3	17.3 ± 3.4
RH (%)	83.8 ± 3.1	78.5 ± 10.2	79.3 ± 12.3	80.5 ± 8.5	83.0 ± 4.3	78.7 ± 10.7	80.4 ± 10.2	80.7 ± 98.4
WS (m/s)	1.4 ± 0.3	1.9 ± 0.8	1.9 ± 0.9	1.7 ± 0.7	1.7 ± 0.5	2.1 ± 0.8	1.8 ± 0.9	1.9 ± 0.7

T: average temperature; RH: average relative humidity; WS: average wind speed.

**Table 2:** Major water-soluble ion concentrations (μg/m^3^) and the corresponding contribution to PM_2.5_ (%), sulphur oxidation ratio (SOR), and nitrogen oxidation ratio (NOR) in PM_2.5_ at the YT and KD sites.

Ions	YT				KD			
	Spring	Autumn	Winter	Average	Spring	Autumn	Winter	Average
PM_2.5_ (μg/m^3^)	61.7 ± 18.5	82.2 ± 30.9	93.4 ± 38.5	79.1 ± 29.3	56.9 ± 19.3	68.2 ± 20.2	95.6 ± 33.5	73.6 ± 24.3
Cl^−^ (μg/m^3^)	1.6 ± 0.6	2.1 ± 0.8	3.8 ± 1.6	2.5 ± 1.0	1.8 ± 0.8	1.6 ± 0.7	3.7 ± 1.6	2.4 ± 1.0
NO_3_^−^ (μg/m^3^)	8.0 ± 3.9	9.0 ± 6.3	14.9 ± 8.3	10.6 ± 6.1	6.3 ± 4.4	7.9 ± 4.5	14.6 ± 6.0	9.6 ± 5.0
SO_4_^2−^ (μg/m^3^)	17.2 ± 4.4	15.6 ± 4.2	14.7 ± 5.4	15.9 ± 4.7	15.1 ± 4.3	14.1 ± 4.3	13.9 ± 4.4	14.4 ± 4.3
NH_4_^+^ (μg/m^3^)	6.9 ± 2.4	5.5 ± 2.0	7.1 ± 3.7	6.5 ± 2.7	4.5 ± 2.6	5.1 ± 2.4	7.1 ± 3.3	5.6 ± 2.7
Cl^−^/PM_2.5_ (%)	2.6 ± 0.9	2.7 ± 1.0	4.3 ± 1.6	3.2 ± 1.3	2.1 ± 0.9	1.8 ± 0.8	4.3 ± 1.8	2.8 ± 1.2
NO_3_^−^/PM_2.5_ (%)	12.4 ± 2.8	11.0 ± 6.6	16.0 ± 5.9	13.2 ± 5.1	9.5 ± 5.11	9.3 ± 5.2	17.1 ± 7.1	12.0 ± 5.8
SO_4_^2−^/PM_2.5_ (%)	28.2 ± 2.1	20.3 ± 5.1	16.5 ± 4.1	21.7 ± 3.8	17.7 ± 5.0	16.6 ± 5.0	16.3 ± 5.1	16.9 ± 5.0
NH_4_^+^/PM_2.5_ (%)	11.1 ± 1.2	6.9 ± 2.1	7.9 ± 3.5	8.6 ± 2.6	5.3 ± 3.1	6.0 ± 2.8	8.3 ± 3.8	6.5 ± 3.2
SIAs	32.1 ± 10.7	30.1 ± 10.0	36.6 ± 16.3	32.9 ± 12.3	27.7 ± 10.5	27.2 ± 9.1	35.6 ± 12.4	30.2 ± 10.6
WSIIs	42.3 ± 12.0	41.2 ± 11.9	50.3 ± 20.2	44.6 ± 14.7	36.3 ± 11.2	36.1 ± 10.0	50.3 ± 16.6	40.9 ± 12.6
SIAs/WSIIs (%)	75.1 ± 4.4	72.3 ± 5.7	71.5 ± 8.5	73.0 ± 6.2	75.3 ± 5.7	74.7 ± 4.0	70.6 ± 6.4	73.5 ± 5.4
WSIIs/PM_2.5_ (%)	68.9 ± 4.6	52.7 ± 12.2	56.0 ± 12.4	59.2 ± 9.8	64.7 ± 9.7	53.8 ± 7.7	54.7 ± 13.8	57.7 ± 10.4
SIAs/PM_2.5_ (%)	51.7 ± 4.5	38.2 ± 9.4	40.4 ± 10.8	43.5 ± 8.3	49.0 ± 9.9	40.2 ± 6.1	38.5 ± 9.6	42.6 ± 8.5
NO_3_^−^/SO_4_^2−^	0.4 ± 0.1	0.6 ± 0.4	1.0 ± 0.5	0.7 ± 0.3	0.5 ± 0.2	0.6 ± 0.4	1.1 ± 0.3	0.7 ± 0.3
SOR	0.3 ± 0.1	0.2 ± 0.1	0.2 ± 0.1	0.3 ± 0.1	0.3 ± 0.1	0.3 ± 0.1	0.3 ± 0.1	0.3 ± 0.1
NOR	0.1 ± 0.0	0.2 ± 0.1	0.2 ± 0.1	0.2 ± 0.1	0.2 ± 0.1	0.2 ± 0.1	0.2 ± 0.1	0.2 ± 0.1

**Table 3:** Seasonal distribution of carbonaceous species over the three seasons at the YT and KD sites.

Carbonaceous	YT				KD			
	Spring	Autumn	Winter	Average	Spring	Autumn	Winter	Average
OC (μg/m^3^)	7.7 ± 1.4	16.2 ± 7.6	16.4 ± 9.3	13.4 ± 6.1	4.6 ± 2.1	13.0 ± 4.2	16.1 ± 9.1	11.2 ± 5.2
EC (μg/m^3^)	4.9 ± 0.8	6.7 ± 2.2	6.4 ± 2.3	6.0 ± 1.8	3.4 ± 1.3	5.0 ± 1.4	5.9 ± 2.2	4.8 ± 1.6
OC/EC	1.6 ± 0.2	2.4 ± 0.7	2.5 ± 1.0	2.1 ± 0.6	1.3 ± 0.2	2.7 ± 0.8	2.7 ± 1.2	2.2 ± 0.8
POC (μg/m^3^)	4.3 ± 0.7	5.9 ± 1.9	5.6 ± 2.0	5.3 ± 1.5	3.5 ± 1.3	5.2 ± 1.4	6.1 ± 2.2	4.9 ± 1.7
SOC (μg/m^3^)	3.4 ± 1.0	10.3 ± 6.0	10.8 ± 7.8	8.2 ± 4.9	1.1 ± 0.9	7.8 ± 3.5	10.1 ± 7.5	8.5 ± 6.5
OC/PM_2.5_ (%)	12.8 ± 2.3	19.8 ± 5.4	16.5 ± 6.1	16.4 ± 4.6	8.1 ± 3.2	19.3 ± 4.3	16.3 ± 6.8	14.5 ± 4.8
EC/PM_2.5_ (%)	8.2 ± 1.2	8.4 ± 1.2	7.1 ± 1.8	7.9 ± 1.4	6.0 ± 1.7	7.5 ± 1.3	6.3 ± 1.5	6.6 ± 1.5
SOC/OC (%)	43.6 ± 7.2	60.7 ± 9.5	56.9 ± 20.4	53.7 ± 12.4	19.8 ± 1.4	57.6 ± 12.9	53.8 ± 20.8	43.7 ± 16.0
SOC/PM_2.5_ (%)	5.7 ± 1.8	12.4 ± 5.3	10.3 ± 6.2	9.2 ± 4.4	1.9 ± 1.7	11.5 ± 4.6	9.8 ± 6.6	7.7 ± 4.3

OC: organic carbon, EC: elemental carbon, POC: primary organic carbon, SOC: secondary organic carbon.

**Table 4:** Concentrations (ng/m^3^) of metal elements in PM_2.5_ during the sampling periods at the YT and KD sites.

Metal	YT				KD			
	Spring	Autumn	Winter	Average	Spring	Autumn	Winter	Average
Mg	117.7 ± 86.3	185.1 ± 126.8	219.2 ± 126.3	174.0 ± 113.2	117.9 ± 74.6	152.1 ± 102.3	200.4 ± 79.4	156.8 ± 85.4
Al	366.7 ± 300.9	451.8 ± 224.5	482.3 ± 264.7	463.6 ± 263.6	351.2 ± 267.0	441.8 ± 221.0	448.6 ± 225.0	413.9 ± 237.7
K	519.1 ± 301.1	1090.2 ± 515.4	1244.6 ± 595.9	951.3 ± 470.8	481.4 ± 329.6	938.1 ± 390.0	1027.4 ± 433.1	815.6 ± 384.2
Ca	136.4 ± 60.3	255.1 ± 171.1	250.8 ± 155.6	214.1 ± 129.0	295.1 ± 279.8	175.5 ± 93.5	237.3 ± 96.5	236.0 ± 156.6
V	2.4 ± 1.8	4.3 ± 1.7	4.1 ± 3.1	3.6 ± 2.2	3.1 ± 1.8	3.3 ± 1.3	3.8 ± 1.4	3.4 ± 1.5
Cr	4.2 ± 2.5	11.7 ± 13.6	10.6 ± 4.1	8.8 ± 6.7	4.1 ± 1.7	5.4 ± 2.3	9.8 ± 4.1	6.4 ± 2.7
Mn	25.2 ± 18.4	35.5 ± 17.4	41.5 ± 25.1	34.1 ± 20.3	20.4 ± 10.5	34.6 ± 19.0	51.8 ± 27.6	35.6 ± 19.0
Fe	925.7 ± 893.8	548.2 ± 246.0	734.8 ± 792.7	736.2 ± 644.2	404.5 ± 297.0	502.0 ± 238.5	710.7 ± 616.5	539.1 ± 384.0
Co	15.5 ± 5.4	23.6 ± 5.3	25.6 ± 9.5	21.5 ± 6.7	16.9 ± 5.4	20.4 ± 3.8	24.9 ± 7.6	20.7 ± 6.0
Ni	3.5 ± 1.4	5.3 ± 2.1	5.5 ± 4.0	4.8 ± 2.5	4.5 ± 2.7	4.6 ± 2.4	4.8 ± 2.3	4.6 ± 2.5
Cu	23.6 ± 17.8	29.0 ± 14.5	38.0 ± 33.2	30.2 ± 21.8	15.3 ± 4.9	27.3 ± 20.3	30.9 ± 26.7	24.5 ± 17.3
Zn	299.6 ± 224.2	361.9 ± 198.0	239.9 ± 204.3	300.5 ± 208.8	294.6 ± 209.9	323.3 ± 255.7	208.0 ± 168.5	275.3 ± 211.3
As	113.7 ± 105.4	71.1 ± 61.2	51.1 ± 42.9	78.6 ± 69.8	28.6 ± 18.5	47.8 ± 32.5	42.9 ± 34.6	39.8 ± 28.6
Se	6.0 ± 3.8	11.0 ± 4.1	11.5 ± 6.9	9.5 ± 4.9	7.9 ± 4.8	13.4 ± 4.0	15.8 ± 7.4	12.4 ± 5.4
Mo	0.7 ± 0.5	1.2 ± 0.7	1.6 ± 1.3	1.2 ± 0.8	0.5 ± 0.3	0.9 ± 0.4	1.2 ± 0.4	0.9 ± 0.4
Cd	4.4 ± 6.3	7.9 ± 7.0	4.9 ± 4.2	5.7 ± 5.8	4.7 ± 5.1	5.8 ± 6.7	5.3 ± 7.6	5.3 ± 6.5
Sb	207.4 ± 51.9	344.2 ± 75.4	412.9 ± 146.6	321.5 ± 91.3	242.9 ± 70.6	293.6 ± 46.5	404.0 ± 116.5	313.5 ± 77.9
Ba	14.1 ± 7.4	33.5 ± 26.5	65.9 ± 68.3	37.9 ± 34.1	18.3 ± 15.6	21.4 ± 11.8	53.8 ± 48.9	31.2 ± 25.4
Tl	0.5 ± 0.5	0.9 ± 0.5	1.4 ± 1.1	0.9 ± 0.7	0.4 ± 0.3	0.8 ± 0.7	0.9 ± 0.6	0.4 ± 0.5
Pb	53.3 ± 49.0	134.4 ± 105.6	155.1 ± 118.1	114.3 ± 90.9	60.4 ± 46.2	105.0 ± 109.4	123.2 ± 107.3	96.2 ± 87.6

## References

[1] Qiao B., Chen Y., Tian M., Wang H., Yang F., Shi G., Zhang L., Peng C., Luo Q., Ding S. (2019). Characterization of water soluble inorganic ions and their evolution processes during PM_2.5_ pollution episodes in a small city in southwest China. Sci. Total Environ..

[2] Tao J., Zhang L., Cao J., Zhang R. (2017). A review of current knowledge concerning PM_2.5_ chemical composition, aerosol optical properties and their relationships across China. Atmos. Chem. Phys..

[3] Shi Z., Krom M.D., Bonneville S., Benning L.G. (2015). Atmospheric processing outside clouds increases soluble iron in mineral dust. Environ. Sci. Technol..

[4] Wang H., Tian M., Chen Y., Shi G., Liu Y., Yang F., Zhang L., Deng L., Yu J., Peng C. (2018). Seasonal characteristics, formation mechanisms and source origins of PM_2.5_ in two megacities in Sichuan Basin, China. Atmos. Chem. Phys..

[5] Tao J., Zhang L., Cao J., Zhong L., Chen D., Yang Y., Chen D., Chen L., Zhang Z., Wu Y. (2017). Source apportionment of PM_2.5_ at urban and suburban areas of the Pearl River Delta Region, south China—With emphasis on ship emissions. Sci. Total Environ..

[6] Yu S., Liu W., Xu Y., Yi K., Zhou M., Tao S., Liu W. (2019). Characteristics and oxidative potential of atmospheric PM_2.5_ in Beijing: Source apportionment and seasonal variation. Sci. Total Environ..

[7] Qi M., Jiang L., Liu Y., Xiong Q., Sun C., Li X., Zhao W., Yang X. (2018). Analysis of the characteristics and sources of carbonaceous aerosols in PM_2.5_ in the Beijing, Tianjin, and Langfang region, China. Int. J. Environ. Res. Public Health.

[8] Wang H., Qiao L., Lou S., Zhou M., Chen J., Wang Q., Tao S., Chen C., Huang H., Li L. (2015). PM_2.5_ pollution episode and its contributors from 2011 to 2013 in urban Shanghai, China. Atmos. Environ..

[9] Gao J., Tian H., Ke C., Long L., Mei Z., Wang S., Hao J., Wang K., Hua S., Zhu C. (2015). The variation of chemical characteristics of PM_2.5_ and PM_10_ and formation causes during two haze pollution events in urban Beijing, China. Atmos. Environ..

[10] Zhang R., Sun X., Huang Y., Shi A., Yan J., Nie T., Yan X., Li X. (2018). Secondary inorganic aerosols formation during haze episodes at an urban site in Beijing, China. Atmos. Environ..

[11] Yang Y., Liu X., Qu Y., An J., Jiang R., Zhang Y., Sun Y., Wu Z., Zhang F., Xu W. (2015). Characteristics and formation mechanism of continuous hazes in China: A case study during the autumn of 2014 in the North China Plain. Atmos. Chem. Phys..

[12] Tang X., Chen X., Tian Y. (2017). Chemical composition and source apportionment of PM_2.5_—A case study from one year continuous sampling in the Chang-Zhu-Tan urban agglomeration. Atmos. Pollut. Res..

[13] Zhang K., Chai F., Zheng Z., Yang Q., Li J., Wang J., Zhang Y. (2014). Characteristics of atmospheric particles and heavy metals in winter in Chang-Zhu-Tan city clusters, China. J. Environ. Sci..

[14] Wang Z., Xu X., Chen R. (2016). Regional distribution characteristics of polycyclic aromatic hydrocarbons in PM_2.5_ and health risk assessment in winter in Xiangtan, Hu’nan. J. Environ. Health.

[15] Xu L., Chen X., Chen J. (2012). Seasonal variations and chemical compositions of PM_2.5_ aerosol in the urban area of Fuzhou, China. Atmos. Res..

[16] Zhang F., Zhao J., Chen J. (2011). Pollution characteristics of organic and elemental carbon in PM_2.5_ in Xiamen, China. J. Environ. Sci..

[17] Liu B., Song N., Dai Q., Mei R., Sui B., Bi X., Feng Y. (2016). Chemical composition and source apportionment of ambient PM_2.5_ during the non-heating period in Taian, China. Atmos. Res..

[18] Paatero P., Tapper U. (1994). Positive matrix factorization: A non-negative factor model with optimal utilization of error estimates of data values. Environmetrics.

[19] Li H., Wang Q., Yang M., Li F., Wang J., Sun Y., Wang C. (2016). Chemical characterization and source apportionment of PM_2.5_ aerosols in a megacity of Southeast China. Atmos. Res..

[20] Liu B., Wu J., Zhang J., Wang L., Yang J., Liang D., Dai Q., Bi X., Feng Y., Zhang Y. (2017). Characterization and source apportionment of PM_2.5_ based on error estimation from EPA PMF 5.0 model at a medium city in China. Environ. Pollut..

[21] Gao J., Peng X., Chen G., Xu J., Shi G., Zhang Y., Feng Y. (2016). Insights into the chemical characterization and sources of PM_2.5_ in Beijing at a 1-h time resolution. Sci. Total Environ..

[22] Ming L., Jin L., Li J., Fu P., Yang W., Liu D., Zhang G., Wang Z., Li X. (2017). PM_2.5_ in the Yangtze River Delta, China: Chemical compositions, seasonal variations, and regional pollution.events. Environ. Pollut..

[23] Fu Q., Zhuang G., Wang J., Xu C., Huang K., Li J., Hou B., Lu T., Streets D. (2008). Mechanism of formation of the heaviest pollution episode ever recorded in the Yangtze River Delta, China. Atmos. Environ..

[24] Gao J., Wang K., Wang Y., Liu S., Zhu C., Hao J., Liu H., Hua S., Tian H. (2018). Temporal-spatial characteristics and source apportionment of PM_2.5_ as well as its associated chemical species in the Beijing—Tianjin—Hebei region of China. Environ. Pollut..

[25] Tan J., Zhang L., Zhou X., Duan J., Li Y., Hu J., He K. (2017). Chemical characteristics and source apportionment of PM_2.5_ in Lanzhou, China. Sci. Total Environ..

[26] Xu H., Xiao Z., Chen K., Tang M., Zheng N., Li P., Yang N., Yang W., Deng X. (2019). Spatial and temporal distribution, chemical characteristics, and sources of ambient particulate matter in the Beijing—Tianjin—Hebei region. Sci. Total Environ..

[27] Al-Naiema I.M., Yoon S., Wang Y., Zhang Y., Sheesley R.J., Stone E.A. (2018). Source apportionment of fine particulate matter organic carbon in Shenzhen, China by chemical mass balance and radiocarbon methods. Environ. Pollut..

[28] Zhang H., Cheng S., Li J., Yao S., Wang X. (2019). Investigating the aerosol mass and chemical components characteristics and feedback effects on the meteorological factors in the Beijing—Tianjin—Hebei region, China. Environ. Pollut..

[29] Ohta S., Okita T.A. (1990). Chemical characterization of atmospheric aerosol in Sapporo. Atmos. Environ..

[30] Zhou J., Xing Z., Deng J., Du K. (2016). Characterizing and sourcing ambient PM_2.5_ over key emission regions in china Ι: Water-soluble ions and carbonaceous fractions. Atmos. Environ..

[31] Tao J., Zhang L., Ho K.F., Zhang R., Lin Z., Zhang Z., Lin M., Cao J., Liu S., Wang G. (2014). Impact of PM_2.5_ chemical compositions on aerosol light scattering in Guangzhou—The largest megacity in South China. Atmos. Res..

[32] Cesari D., Donateo A., Conte M., Merico E., Giangreco A., Giangreco F., Contini D. (2016). An inter- comparison of PM_2.5_ at urban and urban background sites: Chemical characterization and source apportionment. Atmos. Res..

[33] Hsu C., Chiang H., Chen M., Chuang C., Tsen C., Fang G., Tsai Y., Chen N., Lin T., Lin S. (2017). Ambient PM_2.5_ in the residential area near industrial complexes: Spatiotemporal variation, source apportionment, and health impact. Sci. Total Environ..

[34] Morishita M., Gerald J., Keeler G.J., Kamal A.S., Wagner J.G., Harkema J.R., Rohr A.C. (2011). Source identification of ambient PM_2.5_ for inhalation exposure studies in Steubenville, Ohio using highly time-resolved measurements. Atmos. Environ..

[35] Jain S., Sharma S.K., Choudhary N., Masiwal R., Saxena M., Sharma A., Sharma C. (2017). Chemical characteristics and source apportionment of PM_2.5_ using PCA/APCS, UNMIX, and PMF at an urban site of Delhi, India. Environ. Sci. Pollut. Res..

[36] Lee J.H., Yoshida Y., Turpin B.J., Hopke P.K., Poirot R.L., Lioy P.J., Oxley J.C. (2002). Identification of Sources Contributing to Mid-Atlantic Regional Aerosol. J. Air Waste Manage. Assoc..

[37] Banerjee T., Murari V., Kumar M., Raju M.P. (2015). Source apportionment of airborne particulates through receptor modeling: Indian scenario. Atmos. Res..

[38] Yao L., Yang L., Yuan Q., Yan C., Dong C., Meng C., Sui X., Yang F., Lu Y., Wang W. (2016). Sources apportionment of PM_2.5_ in a background site in the North China Plain. Sci. Total Environ..

[39] Pian W., Cheng W., Niu H., Fan J. (2016). TEM study of fine particles from coal-fired power plant ambient air. World J. Eng..

[40] Feng J., Yu H., Mi K., Su X., Li Y., Li Q., Sun J. (2018). One year study of PM_2.5_ in Xinxiang city, North China: Seasonal characteristics, climate impact and source. Ecotoxicol. Environ. Saf..

[41] Meng C., Wang L., Zhang F., Wei Z., Ma S., Ma X., Yang J. (2016). Characteristics of concentrations and water-soluble inorganic ions in PM_2.5_ in Handan city, Hebei province, China. Atmos. Res..

[42] Rengarajan R., Sudheer A.K., Sarin M.M. (2011). Wintertime PM_2.5_ and PM_10_ carbonaceous and inorganic constituents from urban site in western India. Atmos. Res..

[43] Han B., Zhang R., Yang W., Bai Z., Ma Z., Zhang W. (2016). Heavy haze episodes in Beijing during January 2013: Inorganic ion chemistry and source analysis using highly time-resolved measurements from an urban site. Sci. Total Environ..

[44] Huang X., Liu Z., Zhang J., Wen T., Ji D., Wang Y. (2016). Seasonal variation and secondary formation of size-segregated aerosol water-soluble inorganic ions during pollution episodes in Beijing. Atmos. Res..

